# Eligibility of patients withheld or withdrawn from life-sustaining treatment to organ donation after circulatory arrest death: epidemiological feasibility study in a French Intensive Care Unit

**DOI:** 10.1186/2110-5820-3-36

**Published:** 2013-11-07

**Authors:** Olivier Lesieur, Marie-France Mamzer, Maxime Leloup, Frédéric Gonzalez, Alexandre Herbland, Brigitte Hamon, Marcel-Louis Viallard, Christian Hervé

**Affiliations:** 1 Réanimation, Hôpital Saint-Louis, La Rochelle 17019, France; 2 Laboratoire d’Ethique Médicale, Université Paris Descartes, Paris 75006, France

**Keywords:** Tissue and organ harvesting, Withholding treatment, Life support care, Medical futility, Organ donation

## Abstract

**Background:**

Transplantation brings sustainably improved quality of life to patients with end-stage organ failure. Persisting shortfall in available organs prompted French authorities and practitioners to focus on organ retrieval in patients withdrawn from life-sustaining treatment and awaiting cardiac arrest (Maastricht classification category III). The purpose of this study was to assess the theoretical eligibility of non-heart-beating donors dying in the intensive care unit (ICU) after a decision to withhold or withdraw life-sustaining treatment (WoWt).

**Methods:**

We collected the clinical and biological characteristics of all consecutive patients admitted to our ICU and qualified for a WoWt procedure under the terms of the French Leonetti law governing end-of-life care during a 12-month period. The theoretical organ donor eligibility (for kidney, liver, or lung retrieval) of deceased patients was determined *a posteriori* 1) according to routine medical criteria for graft selection and 2) according to the WoWt measures implemented and their impact on organ viability.

**Results:**

A total of 596 patients (mean age: 67 ± 16 yr; gender ratio M/F: 1.6; mean SAPS (Simplified Acute Physiology Score) II: 54 ± 24) was admitted to the ICU, of which 84 patients (mean age: 71 ± 14 yr, 14% of admissions, gender ratio M/F: 3.2) underwent WoWt measures. Eight patients left the unit alive. Forty-four patients presented a contraindication ruling out organ retrieval either preexisting admission (n = 20) or emerged during hospitalization (n = 24). Thirty-two patients would have been eligible as kidney (n = 23), liver (n = 22), or lung donors (n = 2). Cardiopulmonary support was withdrawn in only five of these patients, and three died within 120 minutes after withdrawal (the maximum delay compatible with organ viability for donor grafts).

**Conclusions:**

In this pilot study, a significant number of patients deceased under WoWt conditions theoretically would have been eligible for organ retrieval. However, the WoWt measures implemented in our unit seems incompatible with donor organ viability. A French multicenter survey of end-of-life practices in ICU may help to identify potential appropriate organ donors and to interpret nation-specific considerations of the related professional, legal, and ethical frameworks.

## Background

**Figure 1:**
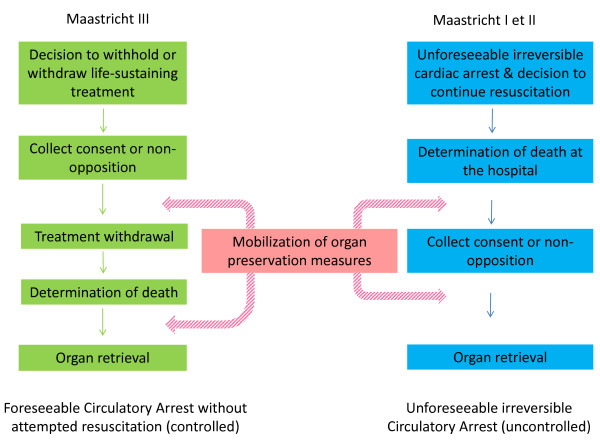
**Organ harvesting under Maastricht I**, **II, and III settings.** The Maastricht classification [5] distinguishes four categories of circulatory arrest death (CAD): unforeseeable irreversible circulatory arrest without (category I) or with (category II) immediate cardiopulmonary resuscitation attempted by trained providers (*uncontrolled* CAD, right side of the panel), foreseeable circulatory arrest occurring after a decision to withhold or withdraw life-sustaining treatment (WoWt) (category III or *controlled* CAD, left side of the panel), circulatory arrest occurring after brain death (category IV, not displayed on the panel).

Qualified as a worldwide shortage, the widening gap between organ demand (i.e., patients in terminal organ failure) and donor graft supply is forcing a rethink of the practical and ethical issues tied to organ transplantation. French policy on organ retrieval essentially hinges on brain dead donors (termed “heart-beating donors”). During the past decade, organ donation following traumatic brain death has become scarcer. Efforts to maintain a pool of available grafts revolved around extending the donor selection criteria to include elderly and/or chronically ill patients (such as diabetics or arterial hypertension sufferers) whose death mostly results from cerebrovascular accidents. This policy seems to have reached its limits and cannot now keep pace with demand. Some countries have developed all or part of their transplantation policy on donation after circulatory arrest [1-4]. In 1995, Dutch transplant surgeons separated out four categories of circulatory arrest death (CAD) into what is known as the Maastricht classification [5]: unforeseeable irreversible circulatory arrest without (category I) or with (category II) immediate cardiopulmonary resuscitation attempted by trained providers (*uncontrolled* CAD), foreseeable circulatory arrest occurring after a decision to withhold or withdraw life-sustaining treatment (WoWt) (category III, *controlled* CAD), circulatory arrest occurring after brain death (category IV). Donations after unforeseeable irreversible circulatory arrest (*uncontrolled* CAD, right-side panel of Figure 1) are authorized in France since 2005 [6]. As the procedure is restricted to a small number of suitably equipped centers, relatively few organs have been retrieved under this system. Organ harvesting in patients deceased under WoWt circumstances (*controlled* CAD, left panel of Figure 1) is still not legally framed in France. The National Academy of Medicine, the National Ethics Advisory Authority (CCNE), and Anesthesia and Critical Care councils had previously voiced concerns over such a procedure arguing that it could be experienced as a form of utilitarian end-of-life practice [7-10].

Recently, a regulatory framework making this type of organ retrieval possible was debated in French parliament [11]. Up to our knowledge, there is no recent epidemiologic data describing French WoWt practices and questioning whether those practices (patient concerned, implemented measures) would be compatible with postmortem organ harvesting. On the basis of other countries experience, it is quite obvious that the period between withdrawal of life-sustaining treatment and death (so-called “the withdrawal period”) is a major determinant of organ donation and of the quality of organs retrieved for transplantation [12]. This period may range from a few minutes to many hours or days, depending on the level of life support engaged at the time of WoWt decision, and how withdrawal is achieved. As clinical guidelines and rules in this area mainly focus on general principles rather than practical details, there is no consensus on the best airway management during the withdrawal period (cessation/reduction of ventilation with/without removal of the endotracheal tube). However, a long withdrawal period often results in severe ischemic damages compromising organ potential use for transplantation [13].

We report the results of a single center retrospective study designed 1) to describe the epidemiological characteristics of patients deceased under a WoWt procedure in a French ICU, 2) to run an *a posteriori* assessment of their theoretical eligibility as organ donors by integrating the measures surrounding WoWt decision and the potential impact of the withdrawal period on organ viability, and 3) to confirm the feasibility of an ongoing French multicenter survey (named “EPILAT”) given IRB approval on 17 December 2012.

## Methods

This pilot study on WoWt practices was carried out in a 16-bed medical-surgical ICU at Saint-Louis Hospital (La Rochelle, France). The institutional review board (CPP Paris Ile de France II, IRB registration: 00001072) approved the protocol (IRB approval n° 2012-11-08). The study observation period ran from November 1, 2011 to October 31, 2012. We retrospectively analyzed the medical records of patients admitted to the ICU who underwent a WoWt procedure in compliance with the terms of the French Leonetti’s law governing end-of-life care [14]. The determinants and procedural conditions governing WoWt decisions follow the French Intensive Care Society guidelines [15]. When current or further life-sustaining treatments appear to be of no overall benefit for a patient, physicians must respect a collegial procedure before making any WoWt decision. The procedure takes into account the patient’s wishes spontaneously expressed or written in advance directives, the opinion of the trusted person, as well as that of the family and close relations. The procedure involves an independent corroboration of the diagnosis and prognosis. In our unit, criteria for withholding or withdrawing treatment are: advanced or terminal stage of a severe and incurable disease, absence of curative strategy, adequate period of time to confirm treatment failure, no additional information needed for decision-making, very high age, limited functional autonomy before hospital admission, limited subsequent functional autonomy, limited subsequent relational quality-of-life, patient’s wish to limit treatment, perception of unreasonable obstinacy voiced by patient’s loved ones [15]. WoWt measures implemented may involve cardiopulmonary resuscitation, ventilatory support, inotropic drugs, dialysis, antibiotics, transfusion, urgent surgery, nutrition, and hydration. Measures agreed on and arguments ensuing collegial debates are recorded in the patient's chart. Patient’s next-of-kin and staff are fully informed. If the patient is still conscious and able to give assent, his agreement to the WoWt features is thoroughly searched and confirmed.

The data recorded on confirmation of death included age, gender, medical history, circumstances surrounding admission, Simplified Acute Physiology Score (SAPS) II index [16] on ICU admission, events occurring during hospitalization, relevant clinical and biological characteristics, Sequential Organ Failure Assessment (SOFA) score [17] at the time of the WoWt decision, modalities governing the WoWt decision (decision-making process, subsequent measures), and outcome of the patient (death or discharged alive). By convention, a SOFA organ subscore of three or more was considered a sign of organ failure.

For patients who died under WoWt conditions, theoretical eligibility as organ donors was determined *a posteriori* based on medical selection criteria, time from withdrawal of life-sustaining treatment to death, and impact of the WoWt measures on organ function. To consider deceased patients as potential donors (regardless of WoWt measures implemented), we excluded those with general contraindications to organ donation (metastatic or hematological malignancy, HIV infection, uncontrolled systemic sepsis, suspected prion disease), and those with absolute organ-elective contraindications to the use of kidney, liver, and lung for transplantation according to the U.K. Guidelines for donation under Maastricht III conditions [18]. Advanced age *per se* was not considered a contraindication. Because hemodynamic parameters during the withdrawal period were irregularly available (due to the retrospective nature of this study), an interval of 2 hours from treatment discontinuation to cessation of cardiac electrical activity was considered the maximum time compatible with organ viability [12,13].

Deidentified when collected, the data are compiled into a spreadsheet and analyzed by the ward statistician. The statistical analysis was descriptive. Continuous variables are reported as means ± standard deviations. Qualitative variables are expressed as absolute values (percentages).

## Results

### Study population

During the study period, 596 patients (age: 67 ± 16 yr; gender ratio M/F: 1.6; SAPS II: 54 ± 24) were admitted to the ICU. Mean length of stay (LOS) was 10 days (minimum 1 day–maximum 122 days). A total of 428 patients (63 ± 16 yr, SAPS II 47 ± 21) survived and were discharged from the ICU after an average LOS of 9 days (minimum 1 day–maximum 97 days). One hundred and sixty-eight patients (28%) died in the ICU: 25 (15%) were registered as brain death and 143 (85%) as circulatory death. Over half the circulatory death patients (76/143, i.e. 53%) underwent a formalized WoWt procedure and subsequently died without resuscitation attempted given the context (*controlled* CAD). For the 67 patients who died of *uncontrolled* CAD, cardiac arrest could not be reversed despite the resuscitation efforts made.

### Context, determinants, and procedural conditions governing the WoWt measures

**Figure 2:**
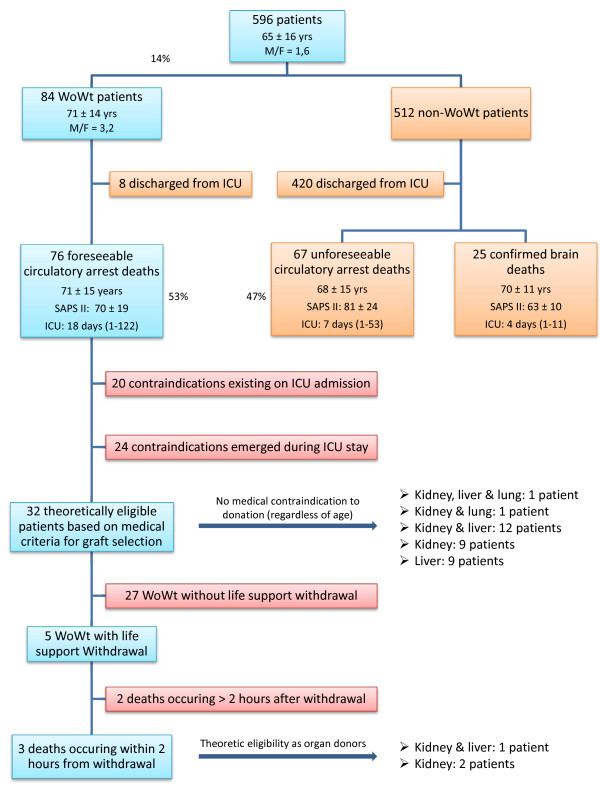
**Flowchart of the 596 patients with regards to the outcome and theoretical eligibility for organ donation.** WoWt, withhold or withdraw treatment; ICU, intensive care unit; SAPS, Simplified Acute Physiology Score.

**Figure 3:**
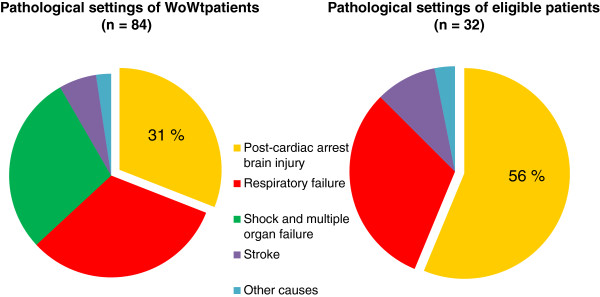
**Pathological settings of WoWt patients and eligible patients for organ harvesting.** Of the 32 patients theoretically eligible as potential donors, 18 had been admitted to the ICU after a successfully resuscitated cardiac arrest and were in persistent postanoxic coma when the collegial WoWt decision was made. This condition counted for close to a third of WoWt patients (left chart) and more than half of the patients theoretically eligible as organ donors before effective WoWt (right chart).

Of the 596 patients admitted, 84 (14%; age: 71 ± 14 yr; gender ratio M/F: 3.2; SAPS II: 69 ± 20) underwent WoWt measures (Figure 2). The circumstances of admission of these patients, as described in the left-side panel of Figure 3, were: acute respiratory distress for 27 patients, out-hospital cardiac arrest successfully resuscitated for 26 patients, shock or multiple organ failure for 24 patients, 5 cases of stroke, 1 encephalitis, and 1 acute kidney failure.

On the day of the WoWt decision, organ failure(s) were distributed as following: respiratory (n = 61 patients), circulatory (n = 43 patients), neurological (n = 38 patients), renal (n = 24 patients), hematological (n = 12 patients), and hepatic (n = 11 patients). On average, patients suffered from 2 to 3 (2.3) organ failures.

Confirmed absence of curative strategy (n = 63 patients), limited subsequent functional autonomy (n = 74 patients), and limited subsequent relational quality of life (n = 65 patients) were the rationales most often put forward to justify WoWt decision.

For eight patients, life-sustaining cardiopulmonary support treatment was withdrawn. Withdrawal of life support usually involved cessation of inotrope/vasoactive drugs and/or discontinuation of ventilatory support. For the other 76 WoWt patients, life-sustaining treatment was limited by measures involving either withholding or progressively phasing out curative therapies. In all cases, comfort care, titrated sedation when needed, hydration, and nutrition were continued.

### WoWt patients’ outcome and theoretical eligibility for organ donation

Of the 84 WoWt patients, 8 survived and were discharged from the ICU, whereas 76 died of circulatory arrest without attempted resuscitation (Figure 2). Mean time from WoWt decision to death was 4.6 days (minimum 0 day–maximum 36 days). Of the eight patients for who life-sustaining cardiopulmonary support was withdrawn, only three died less than 2 hours after treatment withdrawal, four died more than 2 hours later, and one survived and was discharged from the ICU. This comatose, postcardiac arrest patient died 3 days later in a medical ward providing palliative care.

Of the 76 patients who died under WoWt, 20 already presented a contraindication either before or on admission to the ICU that ruled out any organ harvesting (16 metastatic or hematologic malignancies, 3 cases of irreversible multiple organ failure, 1 HIV infection). Twenty-four more patients developed organ dysfunction during their ICU stay (18 cases of multiple organ failure and 6 cases of uncontrolled systemic sepsis). In 4 cases, onset of the organ dysfunction occurred after WoWt measures had been implemented. Therefore, 32 deceased patients (age: 70 ± 15 yr; gender ratio M/F: 2.3; SAPS II: 66 ± 15; SOFA score: 7 ± 3) would ultimately have been eligible for donation of one or more organs based on medical graft selection criteria (regardless of WoWt measures implemented). The original reason these potential donors were admitted to the ICU (Figure 3) was acute respiratory distress (n = 10 patients), out-hospital cardiac arrest successfully resuscitated (n = 18 patients), stroke (n = 3 patients), and encephalitis (n = 1 patient). For 5 of those 32 eligible patients, life-sustaining cardiopulmonary support treatment was withdrawn (without extubation). Only 3 of these 5 patients died less than 2 hours after treatment withdrawal, the timeframe considered compatible with postmortem organ retrieval (Table 1).

**Table 1:** Characteristics of the 32 WoWt patients eligible to organ donation based on medical criteria for graft selection

**Sex**	**Age(yr)**	**Clinical settings**	**SAPS II**	**SOFA**	**Organ(s)theoretically eligible to donation**
F**	76	Stroke	54	8	Kidney & liver
F**	90	End-stage respiratory disease	64	2	Kidney
M**	65	End-stage respiratory disease	27	14	Kidney
M	43	Post-resuscitation coma	61	4	Kidney
M	55	Post-resuscitation coma	65	7	Kidney & liver
F	73	Post-resuscitation coma	96	13	Liver
F	83	Post-resuscitation coma	62	7	Kidney
F	64	Post-resuscitation coma	68	7	Kidney
F	52	Post-resuscitation coma	61	8	Kidney, liver & lung
M	92	Post-resuscitation coma	62	8	Liver
M	73	Post-resuscitation coma	81	7	Liver
M	66	Post-resuscitation coma	59	12	Liver
M	56	Post-resuscitation coma	79	9	Liver
M	55	Post-resuscitation coma	79	7	Kidney
M	62	Post-resuscitation coma	78	10	Kidney & liver
M	82	Post-resuscitation coma	81	7	Kidney & liver
M	46	Post-resuscitation coma	71	7	Kidney & liver
M	54	Post-resuscitation coma	51	7	Kidney & liver
M	80	Post-resuscitation coma	56	8	Kidney & liver
M	79	Post-resuscitation coma	83	11	Kidney & liver
M	62	Post-resuscitation coma	50	5	Kidney & liver
F	93	End-stage respiratory disease	48	9	Liver
F	82	End-stage respiratory disease	70	3	Liver
M	84	End-stage respiratory disease	92	13	Liver
M	82	End-stage respiratory disease	82	5	Liver
M	78	End-stage respiratory disease	36	2	Kidney
M	88	End-stage respiratory disease	63	6	Kidney
M	89	End-stage respiratory disease	50	8	Kidney & liver
M	63	End-stage respiratory disease	84	4	Kidney & liver
F	80	Cerebrovascular accident	56	6	Kidney & liver
M	48	Cerebrovascular accident	69	11	Kidney
M	58	Encephalitis	67	3	Kidney & lung

The first five patients were withdrawn from life support (i.e., inotrope/vasoactive drugs and mechanical ventilation). The first three patients died within 120 minutes from life support withdrawal (**). All other patients underwent either withholding or progressive withdrawal of curative therapies. SAPS II, Simplified Acute Physiology Score II; SOFA, Sequential Organ Failure Assessment.

## Discussion

Every day in ICUs, many patients whose organs could potentially save the life of others die under WoWt conditions. Because donation after CAD is still not, but about to be legally framed in France [11], we designed our study to obtain the largest overview of both WoWt practices (not influenced by any other objective) and patients concerned by such procedures. In this cohort (84 WoWt patients), we *a posteriori* identified a subgroup of 32 deceased patients theoretically eligible for organ donation based on medical criteria for graft selection (regardless of WoWt measures implemented). Because many of these potential donors did not die within an appropriately short time period after life-support withdrawal [12,13], we concluded that our WoWt practices in their current form (i.e., progressive removal of life-support devices) would have been incompatible with further organ harvesting.

Of the 32 patients theoretically eligible as potential donors, 18 (56%) had been admitted to the ICU after a successfully resuscitated cardiac arrest and were in persistent postanoxic coma when the collegial WoWt decision was made (Figure 3). This condition counted for a third of WoWt patients and more than half of the patients theoretically eligible as organ donors before effective WoWt.

### Limitations of the study

The significance of these results remains limited to a single-center, retrospective study conducted on a small population (84 WoWt patients) and a short-lasting period (12 months). However, the epidemiological data collected are comparable to those of previous multicenter studies [19-21] in terms of proportion of ICU patients undergoing WoWt measures (14%) and dying of foreseeable circulatory arrest under WoWt settings (53%).

Our survey mixes up conscious and unconscious patients under WoWt conditions. In the United Kingdom, where the rate of organ harvesting under Maastricht III conditions is one of the highest in the world, the most common diagnoses in donors are severe and irreversible brain injuries [22]. However, conscious patients suffering from irreversible severe diseases with no hope for improvement (for example end-stage respiratory disease, locked-in syndrome, atrophic lateral sclerosis) may request both life-support withdrawal (i.e., turn off mechanical ventilation) and subsequent organ donation if not contraindicated [22-24]. In France, Leonetti’s law applies to conscious or unconscious patients, in end-of-life situation or with irreversible and severely-disabling diseases [14]. As donation under Maastricht III conditions is still not legally framed, we deliberately considered every possible scenario encountered in other public health systems.

It is not the duration *per se* but the hemodynamic profile during the withdrawal period that determines the consequences of warm ischemia on organ viability [12]. However, because of the retrospective nature of this study (not influenced by any ulterior motive), hemodynamic parameters during the withdrawal period were rarely available. Moreover, troublesome monitoring often was switched off to leave the patient and relatives peaceful. We arbitrarily considered that an interval of 2 hours from treatment withdrawal to cessation of cardiac electrical activity was the maximum withdrawal period compatible with an hypothetical organ harvesting [12,13].

As organ donation under Maastricht III settings is still not legally framed in France, this study does not address the question of patient/family consent. We keep in mind that the rate of refusal would significantly impact the amount of potential donors proceeding to donation.

### Conditions governing withdrawal of life-sustaining support and time to cardiac death

The WoWt procedures formalized in this study were always established through a systematic, daily collective discussion designed to determine the most appropriate level of care for each hospitalized patient. End-of-life care in France is legally framed under the French law 2005–370 of April 22, 2005 relating to Patients’ rights and to the end of life (so called Leonetti’s law), which authorizes the withholding or withdrawal of curative therapies when deemed “*useless*, *disproportionate or to have no other effect than solely the artificial preservation of life*” [14]. Continuing such treatment with no hope of cure or benefit would equate to an unreasonable obstinacy, especially for patients who are no longer able to express their wishes. In this setting, the law stipulates that any WoWt decision may only be taken after a formal procedure of collegial debates. Obtaining an external opinion from an independent consultant is a compulsory part of the procedure. In addition to medical factors, the Leonetti’s law stipulates that the discussions must integrate the patient’s wishes voiced or written in advance directives, the opinion of the trusted person (if appointed), as well as that of the family and close relations. All proceedings and decisions taken during discussions are recorded in the patient’s medical file. Despite the collegial nature of the procedure, the physician in charge of the patient remains liable for the final decision. Once a life-support withdrawal decision has been made, delivering comfort care becomes priority. While the technical environment of our ICU does not offer optimal conditions for a quiet end-of-life, therapies from this point exclusively focus on relieving pain, anxiety, and discomfort—*what needs to be done when there is nothing more to be done*.

Because circulatory arrest must occur after a short period, only the withdrawal of life-sustaining cardiopulmonary support for highly-dependent patients (high inspired oxygen fraction (FiO2), nontriggered modes of ventilation, inotrope/vasoactive drug use) is compatible with postmortem organ donation [12,13,25-27]. In our study, cardiopulmonary support was withdrawn in 8 of the 84 WoWt patients, 3 of whom died within the first 120 minutes of withdrawal (the maximum delay compatible with organ viability and graft quality for potential donor grafts). We registered a single episode of extubation in 1 of the 84 patients who finally presented no clinical sign of obstruction. This postanoxic comatose patient was given scopolamine and methylprednisolone prior to extubation. Discharged from the ICU, he died 3 days later in a medical ward providing palliative care. Seven additional WoWt patients discharged from the ICU finally left the hospital alive, thus confirming the intention of WoWt measures, which is to let nature take its course (given the irreducible uncertainty about prognosis for critically ill patient) without searching to hasten death. Indeed, there is significant variation in how treatment withdrawals are implemented in ICUs, particularly with regards to airway management. Published guidelines mainly focus on the decision-making principles rather than practical details about how end-of-life care should be managed. Once artificial breathing support is switched off, it becomes possible to remove the endotracheal tube that connects the patient to the ventilator and secures the airway [10]. Rather than an abrupt “on-off” discontinuation of mechanical ventilation, with or without extubation, many teams prefer a progressive withdrawal of mechanical ventilation (termed “terminal weaning”), because they feel the physical symptoms of airway obstruction may harm the patient and be distressing to relatives and caregivers [28]. However, some consider this progressive weaning as an unnecessarily prolonged agony if death is the only possible outcome [29].

### Ethical dilemma between the responsibility to deliver the best care to the dying patient and the need to harvest his organs

This study was led under conditions enabling a state-of-play of ongoing practices without the physicians responsible for WoWt decisions being pressured by any moral dilemma between the individual interest of the dying patient and the collective benefit for potential graft recipients. Although not expressly forbidden under current French law, organ harvesting after controlled circulatory death is still not practiced “*so as to rule out any potential tension between the decision to withdraw treatment and the intention to harvest organs*” [7]. In other countries, teams involved in organ retrieval after controlled death consider organ donation as a routine part of end-of-life care once it is established that the patient wishes to be a donor [10,22,30,31]. Of the 32 patients theoretically eligible as organ donors before effective WoWt measures in our study, 22 presented severe brain lesions and were *de facto*, unable to express their wishes. Because organ donation under Maastricht III conditions is still not practiced in France, patient’s relatives were never asked to provide their loved one’s position on this particular question. Moreover, the medical records before ICU admission made no mention of anticipated directives or of a legally designated trusted person.

Enrolling death into an organ harvesting procedure would entail a number of organizational constraints that may interfere with the comfort care given to the dying patient and their loved ones. In countries that already authorize organ donation after CAD, in order to meet the time framework tied to organ viability, life-support is withdrawn either in the operating room or in the ICU provided that the patient can be swiftly transferred to the operating room once death certified [22]. These operational requirements contrast with the regular palliative approach (i.e., with no intention of organ harvesting). Even though it is theoretically possible to maintain contact between the patient and relatives up to surgical intervention, the technical environment of an operating room is far from the ideal place to organize spiritual assistance and end-of-life rituals [32]. Furthermore, the quality of the organs harvested under such conditions is closely dependent on how early technical organ preservation measures are implemented. One of these technical measures consists in catheterizing the aorta and inferior vena cava in order to connect an extracorporeal pump and maintain circulation in the abdominal organs. Once futile treatments get discontinued according to a formal collegial debate, any intrusive intervention practiced before the subject is declared dead could be seen as conflicting with efforts to deliver terminal comfort care. Moreover, such a procedure could even become intolerable for relatives and care staff if the eventuality of a prolonged agonal period making organ retrieval impossible has not been explicitly addressed beforehand. It is thus essential to precisely predict time to cardiac death after withdrawal of life-sustaining treatment [13,25,27]. Any patient for which the elective WoWt measure is not withdrawal of life-sustaining treatment therefore should be definitively excluded from any intention to retrieve organs.

## Conclusion

In this single-center study, a significant number of patients who died under WoWt procedures would have theoretically been eligible for organ harvesting based on routine medical criteria for graft selection. However, WoWt measures implemented in our ICU are incompatible with this type of organ retrieval, as the prolonged agonal period would irreversibly compromise organ quality and graft viability. A multicenter study performed under the same conditions has been launched in the first half of 2013 to characterize WoWt practices in France and to evaluate the organ donor eligibility of patients dying under such conditions. It thus seems crucial to focus on the factors determining how and when life support has to be withdrawn, particularly discontinuing mechanical ventilation and removing the endotracheal tube. Finally, a sensitive issue may arise in a near future: in which context are we medically and ethically entitled to revise our practices and make them suitable for donation after controlled circulatory arrest death?

## Competing interests

The authors declare that they have no competing interests.

## Authors’ contributions

OL, MM and CH designed the study. OL, ML and AH gathered the data. OL and ML performed the statistical analysis. OL, MFM, FG, BM, MLV and DM wrote the manuscript. OL had full access to all data in the study and had final responsibility for the decision to submit for publication. All authors read and approved the manuscript.
